# Computational Approaches and Observer Variation in the 3D Musculoskeletal Modeling of the Heads of *Anolis*

**DOI:** 10.1093/iob/obae009

**Published:** 2024-05-01

**Authors:** A D Lagorio, F R McGechie, M G Fields, J Fortner, E Mackereth, C Perez, A T Wilken, M Leal, C V Ward, K M Middleton, C M Holliday

**Affiliations:** Department of Pathology and Anatomical Sciences, University of Missouri, Columbia, MO 65212, USA; Department of Basic Medical Sciences, University of Arizona College of Medicine-Phoenix, Phoenix, AZ 85004, USA; Department of Pathology and Anatomical Sciences, University of Missouri, Columbia, MO 65212, USA; Department of Pathology and Anatomical Sciences, University of Missouri, Columbia, MO 65212, USA; Department of Pathology and Anatomical Sciences, University of Missouri, Columbia, MO 65212, USA; Division of Biological Sciences, University of Missouri, Columbia, MO 65211, USA; Department of Organismal Biology and Anatomy, University of Chicago, Chicago, IL 60637, USA; Division of Biological Sciences, University of Missouri, Columbia, MO 65211, USA; Department of Pathology and Anatomical Sciences, University of Missouri, Columbia, MO 65212, USA; Division of Biological Sciences, University of Missouri, Columbia, MO 65211, USA; Department of Pathology and Anatomical Sciences, University of Missouri, Columbia, MO 65212, USA

## Abstract

High-resolution imaging, 3D modeling, and quantitative analyses are equipping evolutionary biologists with new approaches to understanding the variation and evolution of the musculoskeletal system. However, challenges with interpreting DiceCT data and higher order use of modeled muscles have not yet been fully explored, and the error in and accuracy of some digital methods remain unclear. West Indian *Anolis* lizards are a model clade for exploring patterns in functional adaptation, ecomorphology, and sexual size dimorphism in vertebrates. These lizards possess numerous jaw muscles with potentially different anatomies that sculpt the adductor chamber of the skull. Here we test approaches to quantifying the musculoskeletal shape of the heads of two species of *Anolis*: *A. pulchellus* and *A. sagrei*. We employ comparative approaches such as DiceCT segmentation of jaw muscles, 3D surface attachment mapping, and 3D landmarking with the aim of exploring muscle volumes, 3D muscle fiber architecture, and sexual dimorphism of the skull. We then compare sources of measurement error in these 3D analyses while also presenting new 3D musculoskeletal data from the *Anolis* feeding apparatus. These findings demonstrate the accessibility and repeatability of these emerging techniques as well as provide details regarding the musculoskeletal anatomy of the heads of *A. pulchellus* and *A. sagrei* which show potential for further research of comparative biomechanics and evolution in the clade.

## Introduction

Digital imaging and three-dimensional (3D) analysis have become integral to the study of comparative biomechanics and evolutionary biology. The ability to discover and disseminate fine details of vertebrate anatomy has historically relied upon destructive techniques such as serial histological sectioning and gross dissection. In contrast, combinations of modern imaging techniques such as diffusible iodine-based contrast-enhanced computed tomography (DiceCT; Gignac et al. 2016) and 3D digital reconstruction enable the identification of many soft tissue structures without irreversible damage to specimens (Metscher 2009; Holliday et al. 2013; Jones et al. 2019; Hadden et al. 2022). This new coupling of digital imaging modalities with 3D modeling techniques has enabled the measurement of brain volumes (Gignac and Kley 2018), nerve branching patterns (Lessner and Holliday 2020), lung structure (Brocklehurst et al. 2018), and in particular, muscle volumes (Holliday et al. 2013; Cunningham et al. 2014; Sellers et al. 2017; Dickinson et al. 2018; Orsbon et al. 2018; Herbst et al. 2022). Currently it is possible to not only capture the volumes of muscles, but to also model the 3D architecture of muscle fascicles within a muscle belly (Kupczik et al. 2015; Dickinson et al. 2019; Sullivan et al. 2019; Katzke et al. 2022, Holliday et al. 2022), offering the potential of new, more precise estimates of physiological cross-sectional area, and ultimately, estimates of muscle force.

Gaining familiarity with cross-sectional imaging data, such as those produced by histology or computed tomography, remains challenging. This fact is particularly true for soft tissues. Even seasoned anatomists experience some level of difficulty teasing apart muscle bellies from one another in cross-section, where in dissection, clear fascial boundaries might be more easily seen with white light and kinesthetic approaches using instruments (Hadden et al. 2022). Any errors in assessing cross-sectional imaging data such as the misidentification of fascial boundaries or separations of muscle bellies could cascade upwards into higher order calculations of volumes, centroids of attachments, or 3D muscle resultant vectors, and ultimately, comparative evolutionary studies.

Given that the combination of digital cross-sectional imaging methods with 3D modeling techniques is relatively new, it requires more understanding of where sources of variation and error might arise. In particular, statistical noise produced by variation in 3D analyses can have negative impacts on related future analyses. By assessing the variation that produces differences between observers it may be possible to optimize future study designs and help mitigate errors in the quantification of morphological phenotypes (Loken and Gelman, 2017; Vrdoljak et al. 2020). Combined with estimates of measurement error and variability, we aim to pinpoint sources of variation in 3D analyses to better understand how to reduce such statistical noise and improve future repeatability.

Previous studies have used 3D analysis techniques to examine the heads of lizards, particularly with regard to sexual dimorphism (Kaliontzopoulou et al. 2007) and ecomorphology (Kaliontzopoulou et al. 2010). West Indian anoles are small-sized lizards and are, like Darwin's finches of the Galapagos Islands, a model organism for the study of adaptive radiation (Losos et al. 2006; Losos 2009; Burress and Muñoz 2022). *Anolis* ecomorphs are characterized by their microhabitat use and morphology (Williams et al. 1983; Losos 2009). *Anolis pulchellus*, a grass-bush ecomorph, has a small snout-vent length. In contrast, *A. sagrei*, a trunk-ground ecomorph, has a relatively larger snout-vent length (Gorman and Harwood 1977; Legreneur et al. 2012). Both ecomorphs exhibit some degree of sexual dimorphism, thought to be associated with male intrasexual competition (Calsbeek and Marnocha 2006; Losos 2009; Sanger et al. 2013). *Anolis pulchellus* and *A. sagrei* are generalized insectivores but can opportunistically consume small vertebrates (Gorman and Harwood 1977; Norval et al. 2010; Ingram et al. 2022). They possess numerous jaw muscles with potentially varied anatomies that sculpt the adductor chamber of the skull and impact cranial performance (Aerts et al. 1997; Daza et al. 2011; Wittorski et al. 2016). Describing the musculoskeletal elements of the feeding apparatus in these two species using emerging 3D techniques could not only help identify sources of extrinsic measurement error in the assessment of digital imaging data, but begin to shed some light on the relationship between cranial shape and musculoskeletal performance in anoles.

Here, we share the experience of an academically diverse class of University of Missouri graduate students who learned a series of imaging and data analysis tools while using a novel sample of *Anolis* lizards as part of an online course entitled *3D Imaging and Computational Biology for Research Applications*. This course was developed during COVID quarantine as a means of training a cohort of graduate students simultaneously in digital anatomy and computational methods, using largely virtual instruction. Using fully digital 3D modeling and computational approaches, the students were taught interpretation and segmentation of cross-sectional microCT (µCT) and DiceCT data into muscle volumes, 3D surface maps of muscle attachments, and landmark-based geometric morphometrics, among other quantification, visualization, and dissemination techniques. Our findings were analyzed with the aim of exploring sources of measurement error and their potential impact on repeatability. These findings were also examined for their implications in biological inferences including the influence of sexual dimorphism on the skulls of anoles and the visualization of muscle fiber architecture in 3D. We hypothesized that for each set of data being analyzed, any major variations in metric data would more likely be attributable to real anatomical differences exhibited by the *Anolis* specimens than to 3D delineation and modeling errors introduced by the student investigators. While we were limited in numbers of students, computers, and software seats, thereby limiting the scope and power of statistical analysis, these data are quite large in sample compared to most other digital anatomy studies and clearly demonstrate the error and accuracy of these emerging digital techniques while also providing insights on the musculoskeletal anatomy of the heads of two West Indian anole species.

## Methods

### Course Overview

Five graduate students from the Department of Pathology and Anatomical Sciences and the Division of Biological Sciences at the University of Missouri, USA, participated in the course PTH_AS 8285 (*3D Imaging and Computational Biology for Research Applications*), spanning both the Fall and Spring semesters of the 2020–2021 academic year. The students (co-authors AD Lagorio, MG Fields, J Fortner, E Mackereth, and C Perez) and instructors (CM Holliday, FR McGechie, and KM Middleton) came from a wide range of academic experiences possessing varying degrees of prior education, including time spent in graduate school, anatomy background, computational methods background, as well as various levels of expertise in jaw muscle anatomy, *Anolis* life history, CT scan interpretation, and 3D modeling. All students received identical, synchronous instruction via remote video conference calls once weekly in addition to supplemental, asynchronous instruction as needed. Data collection was conducted remotely. Following completion of the course, all data produced were analyzed in terms of anole head shape and measurement of error.

### Anolis pulchellus

#### microCT imaging

**Fig. 1:**
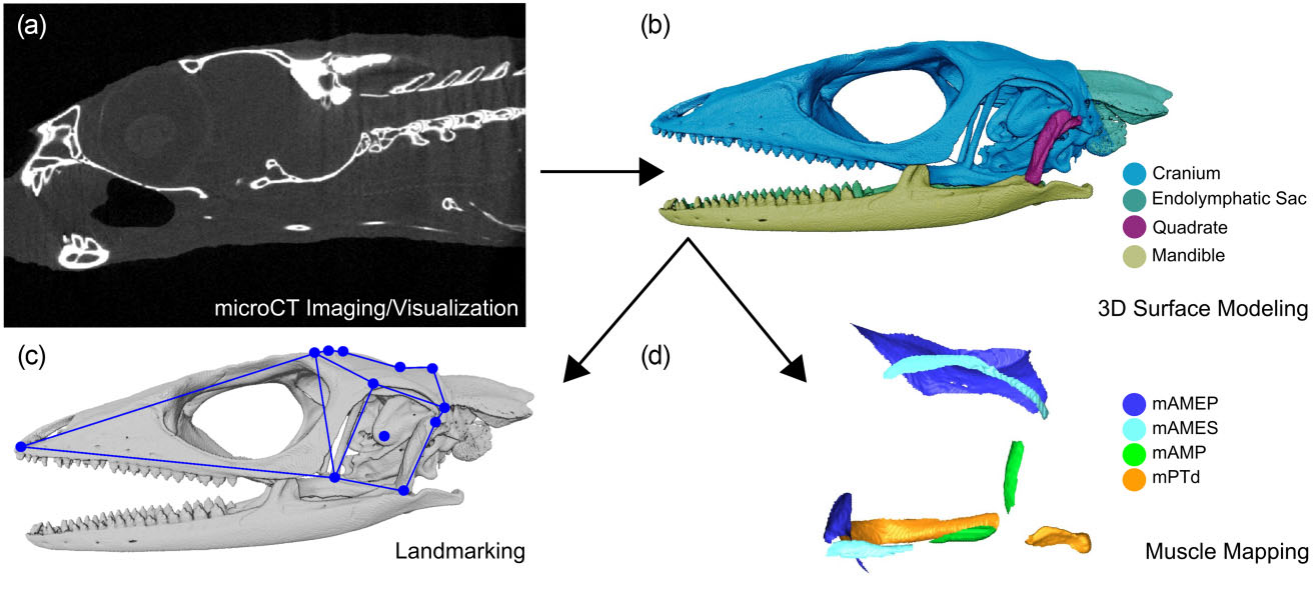
Steps involved in *Anolis pulchellus* skeletal imaging and analysis, outlined using specimen MUVC GY09. (a) Visualization of µCT dataset in left lateral view. (b) 3D surface model consisting of bony skull elements. (c) Novel collection of surface landmarks placed on the cranium of the skull. (d) Isolated surface maps of four jaw muscle attachments. Abbreviations: m. adductor mandibulae externus profundus (mAMEP), m. adductor mandibulae externus superficialis (mAMES), m. adductor mandibulae posterior (mAMP), m. pterygoideus dorsalis (mPTd). These abbreviations apply to all subsequent figures.

Six fresh-frozen cadaveric *A. pulchellus* specimens, three male (MUVC GM06, GM09, PN14) and three female (MUVC GY09, GM10, GM15), were donated to the University of Missouri Vertebrate Collections (MUVC) for use in this study (ACUC #13240; ACUC #10114). Specimens were fixed in a 10% formalin solution for 4 weeks and then underwent µCT scanning at the University of Missouri X-ray Microanalysis Core (MizzoµX) using an Xradia 510 Versa X-ray microscope. Scans were performed at voxel sizes varying from 17.011 to 19.97 µm^3^ (Table 1). Scan data were imported as stacked .tiff files into Avizo v9.1 (Thermo Fisher Scientific) and cropped to only include cranial data (Fig. 1a).

**Table 1:** Imaging parameters of lizard specimens used in this study. MUVC (University of Missouri Vertebrate Collections).

Specimen	Taxon	Sex	Scan type	Pixel size (µm)	kV	mA	Head length (mm)
MUVC PN14	*Anolis pulchellus*	M	µCT	18.17	70	85	8.12
MUVC GM06	*Anolis pulchellus*	M	µCT	19.97	70	85	7.37
MUVC GM09	*Anolis pulchellus*	M	µCT	18.17	70	85	9.17
MUVC GY09	*Anolis pulchellus*	F	µCT	17.01	70	85	8.68
MUVC GM10	*Anolis pulchellus*	F	µCT	18.17	70	85	8.72
MUVC GM15	*Anolis pulchellus*	F	µCT	19.97	70	85	7.57
MUVC LI100	*Anolis sagrei*	M	DiceCT	28.05	70	85	8.60

#### Muscle mapping

Six investigators (five students plus instructor CM Holliday) were assigned their own *A. pulchellus* specimen and tasked with manually segmenting out the bony elements from the cropped heads using Avizo. This resulted in 3D surface models of each scanned *A. pulchellus* skull (Fig. 1b). Students then mapped the sites of attachment of four jaw muscles [m. adductor mandibulae externus superficialis (mAMES), m. adductor mandibulae externus profundus (mAMEP), m. adductor mandibulae posterior (mAMP), and m. pterygoideus (mPT) (Fig. 1d)] on the skull of *A. pulchellus*, assisted by an illustration provided to guide them in this activity. The origin and insertion of all four muscles were mapped onto the left side of each of the six skulls (one skull per student plus one instructor). This process was repeated by two pairs of investigators (each pair consisting of one student and one instructor), each using a single skull, to determine the reproducibility of these methods (one pair produced replicate maps for MUVC GM06, the other produced replicate maps for MUVC GM15). The resulting data were used to produce comparative estimates of muscle volume via frustrum modeling (see Wilken et al. 2019) as well as muscle resultant orientation, which was projected into ternary space (see Cost et al. 2022). Variation of estimated muscle volumes was compared using the coefficient of variation (standard deviation divided by the mean), which is scale-independent. The repeatability of muscle volumes derived from surface maps was calculated as the intraclass correlation coefficient (ICC; Lessels and Boag 1987; Wolak et al. 2012 and referenced therein). The ICC is a measure of interobserver reliability that can be interpreted on a scale from 0 to 1 (Koo and Li 2016). Zero indicates very little consistency between observers, whereas 1 indicates high repeatability. ICC was calculated using the icc() function in the performance package (Lüdecke et al. 2021) based on a mixed model ANOVA with muscle as a random effect using the lmer() function in the lme4 package (Bates et al. 2015) in R. Specifically, we fit the mixed model: muscle volume ∼ 1 + (1 | Muscle ID), which fits an overall intercept with a random intercept for each muscle, where the five individual segmenters are represented once for each muscle. ICC was calculated separately by sex (n = 3 per sex) to assess observer challenges resulting from sexual dimorphism in *A. pulchellus.*

#### Landmark analysis

**Fig. 2:**
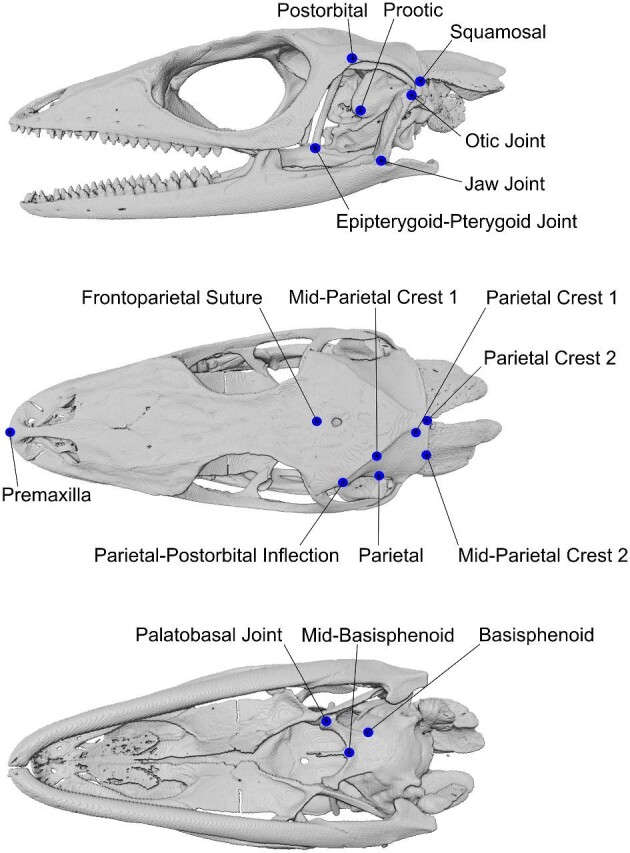
Landmarks (n = 30) included in the 3D GM analysis of *Anolis pulchellus* specimens. Specimen shown is MUVC GY09. Landmarks were collected on both sides of the skull, targeting the adductor chamber, parietal region, premaxilla, and basisphenoid.

The 3D skull models of *A. pulchellus* previously produced in the section “Muscle mapping” (n = 6) were exported from Avizo and used in the identification of surface landmarks (each of the five students plus instructor FR McGechie landmarked all six skulls, Fig. 1c) using Checkpoint software (Stratovan Corporation). Following a schematic that illustrated the general location of morphological landmarks on the skull, the students plus the single instructor placed 30 landmarks targeting the adductor chamber, parietal region, premaxilla, and basisphenoid (Fig. 2). The landmarks included both sides of each skull and were combined to create wireframe models for comparison across specimens that were visualized in Morphologika (O'Higgins and Jones 1998).

Interobserver variation between landmarks was analyzed using a Generalized Procrustes Alignment (see Bookstein 1986; Rohlf and Slice 1990; Shearer et al. 2017) and a measurement of error was calculated as the intraclass correlation coefficient using the ICC function in R (Wolak et al. 2012). A two-way random effect model, for interrater reliability and a measure of consistency was evaluated using an intraclass correlation coefficient (following Koo and Li 2016). To estimate ICC, we calculated pairwise 3D distances among all landmarks for each trial (n = 6 observers, n = 6 specimens, and n = 30 landmarks for a total of 15,660 interlandmark distances). Then, an ICC for each landmark pair was calculated across all trials. Finally, the mean estimate of ICCs was estimated for all landmark pairs across all trials.

Variation in skull shape was analyzed by a principal components analysis in R (R Core Team 2022) using the packages geomorph (Baken et al. 2021) and Euclidean distance matrix analysis (Lele 1991; Solymos et al. 2021) using the package EDMAinR.

### Anolis sagrei

#### DiceCT imaging

**Fig. 3:**
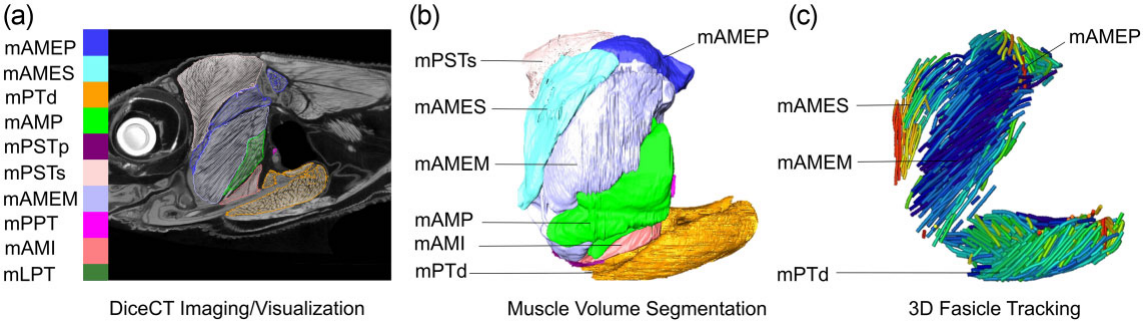
Steps involved in *Anolis sagrei* (MUVC LI100) soft tissue imaging and analysis. (a) DiceCT dataset in left lateral view of parasagittal section. Manually segmented jaw muscles are indicated by individually colored outlines. (b) 3D muscle volumes rendered from segmented DiceCT data. (c) Model of 3D muscle fascicles with color denoting fiber orientation relative to rostrocaudal axis of the head. Abbreviations: m. adductor mandibulae externus medialis (mAMEM), m. pseudotemporalis superficialis (mPSTs), m. pseudotemporalis profundus (mPSTp), m. levator pterygoideus (mLPT), and m. protractor pterygoideus (mPPT). These abbreviations apply to all subsequent figures.

One male fresh-frozen cadaveric specimen of *A. sagrei* (MUVC LI100) was used in this study (ACUC #10114). This specimen was fixed in a 10% formalin solution for 4 weeks. The fixed specimen was immersed in 3% iodine potassium iodide (i.e., Lugol's Iodine) for 8 weeks. After diffusion via immersion was complete, the specimen was taken to and scanned at MizzoµX (Table 1). The resulting DiceCT data were imported as stacked .tiff files into Avizo v9.1 and trimmed to only include elements pertaining to the head (Fig. 3a).

#### Muscle volume segmentation

Using identical copies of the DiceCT data produced from *A. sagrei* specimen MUVC LI100, six investigators (five students plus instructor CM Holliday) manually segmented the muscles of the left adductor chamber (Fig. 3b). These included m. adductor mandibulae externus medialis (mAMEM), m. adductor mandibulae externus profundus (mAMEP), m. adductor mandibulae externus superficialis (mAMES), m. adductor mandibulae posterior (mAMP), m. pterygoideus dorsalis (mPTd), m. pseudotemporalis superficialis (mPSTs), m. pseudotemporalis profundus (mPSTp), m. levator pterygoideus (mLPT), and m. protractor pterygoideus (mPPT). The students were guided through identifying fascial planes and boundaries between particular muscles, providing starting points in some cases. However, mPSTs and mAMEP, two muscles whose boundaries are difficult to interpret, were largely left to the students to interpret on their own. As for *A. pulchellus*, variation of estimated muscle volumes was compared using the coefficient of variation. The repeatability of segmented muscle volumes was calculated as ICC (Wolak et al. 2012). ICC was calculated using the function icc() in the performance package (Lüdecke et al. 2021) as described above for *A. pulchellus*.

#### Muscle mapping

Bony elements of the *A. sagrei* (MUVC LI100) skull were manually segmented from the DiceCT data in Avizo. The sites of attachment of four jaw muscles mAMES, mAMEP, mAMP, and mPT, were mapped onto the surface model of the skull. For each muscle, a combination of surface area and centroid data were used to produce estimates of muscle volume using frustrum modeling (see Wilken et al. 2019), which were compared against the previously segmented muscle volumes produced in the section “Muscle volume segmentation”. The variation between the two methods of estimating muscle volumes was compared using the coefficient of variation.

#### Muscle fascicle architecture

The segmented muscles from *A. sagrei* specimen MUVC LI100 produced by instructor CM Holliday in the section “Muscle volume segmentation” were selected for 3D fascicle tracking using the machine learning algorithm Avizo XFiber. Xfiber automatically segments out perceived individual fascicles within each muscle and can colorize their angular relationships relative to the long axis of the muscle belly using the “Orientation_Theta” data visualization tool (Fig. 3c). This process required training Xfiber to recognize individual muscle fascicles through the creation of a cylindrical template (see Sullivan et al. 2019) which was then propagated through the segmented portion of the DiceCT cross-sectional data, ultimately producing traced fascicle tracks representative of each muscle's internal architecture. The resulting data were exported from Avizo and used to produce ternary plots illustrating the individual fascicle alignment throughout each muscle in R using the package MuscleTernary (Middleton 2022).

## Results

### Anolis pulchellus

#### Muscle mapping

**Fig. 4:**
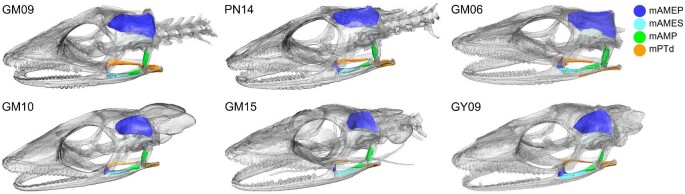
Surface mapped muscle attachments in six specimens of MUVC *Anolis pulchellus*. Males (n = 3) on top and females (n = 3) on bottom.

**Fig. 5:**
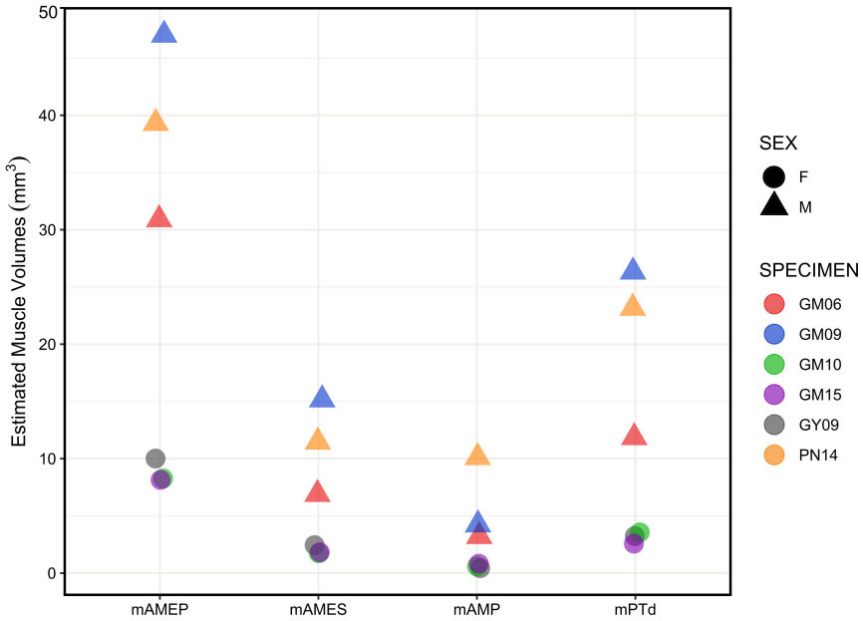
Estimates of jaw muscle volumes for *Anolis pulchellus*. 3D muscle volumes estimated from a combination of surface muscle maps and centroid placements across six specimens of *A. pulchellus*. The greatest variation occurs between the sexes, with males averaging greater combined muscle volume estimates than females (see Table 3).

There were no observable differences in the shapes and locations of muscle maps produced for the jaw muscles of *A. pulchellus* (n = 6, Fig. 4). However, muscle volumes differed between the sexes, with males averaging larger volumes for all muscles compared to females (Fig. 5). mAMEP had the greatest average volume estimate across all six skulls, followed by mPTd, mAMES, and mAMP, which had the smallest (Tables 2 and 3). Coefficient of variation ranged from 11.8% to 33% in females and from 20.6% to 63.9% in males. This general trend toward more variation in males was reflected in ICC. ICC for muscle volume estimated from mapped muscle attachment areas was 0.97 for females and 0.84 for males. These patterns were confirmed when investigators of varying expertise (student vs. instructor) were tasked with mapping replicate surface attachments for all four muscles onto copies of the same skull, which yielded similar results (Tables 4 and 5). The duplicate trials produced a coefficient of variation ranging from 1.8% to 34.8% in GM15 (female) and from 6.8% to 56.0% in GM06 (male).

**Table 2:** Estimated muscle volumes produced via the surface mapping and frustrum modeling of four jaw muscles across six specimens of *A. pulchellus*.

Investigator	Specimen (MUVC)	Sex	Muscle	Volume (mm^3^)
1	PN14	M	mAMES	11.46
1	PN14	M	mAMEP	39.31
1	PN14	M	mAMP	10.13
1	PN14	M	mPTd	23.14
2	GY09	F	mAMES	2.45
2	GY09	F	mAMEP	10.00
2	GY09	F	mAMP	0.41
2	GY09	F	mPTd	3.21
3	GM09	M	mAMES	15.13
3	GM09	M	mAMEP	47.01
3	GM09	M	mAMP	4.21
3	GM09	M	mPTd	26.31
4	GM06	M	mAMES	6.90
4	GM06	M	mAMEP	30.89
4	GM06	M	mAMP	3.21
4	GM06	M	mPTd	11.84
5	GM10	F	mAMES	1.74
5	GM10	F	mAMEP	8.28
5	GM10	F	mAMP	0.60
5	GM10	F	mPTd	3.54
6	GM15	F	mAMES	1.80
6	GM15	F	mAMEP	8.13
6	GM15	F	mAMP	0.81
6	GM15	F	mPTd	2.56

**Table 3:** Mean muscle volumes (mm^3^) and coefficient of variation produced via the surface mapping and frustrum modeling of four jaw muscles across six *A. pulchellus* specimens. (A) Male specimens (MUVC PN14, GM06, GM09). (B) Female specimens (MUVC GY09, GM10, GM15).

	A	mAMES	mAMEP	mAMP	mPTd	B	mAMES	mAMEP	mAMP	mPTd
		11.46	39.31	10.13	23.14		2.45	10.00	0.41	3.21
		15.13	47.01	4.21	26.31		1.74	8.28	0.60	3.54
		6.90	30.89	3.21	11.84		1.80	8.13	0.81	2.56
Mean		11.16	39.07	5.85	20.43		2.00	8.80	0.61	3.10
Standard deviation		4.12	8.06	3.74	7.61		0.39	1.04	0.20	0.50
Coef. of variation		36.9	20.6	63.9	37.2		19.7	11.8	33.0	16.1

**Table 4:** Estimated muscle volumes produced by investigators of varying expertise via the repeated surface mapping and frustrum modeling of four jaw muscles across two *A. pulchellus* specimens.

Investigator	Specimen (MUVC)	Sex	Muscle	Volume (mm^3^)
Student	GM06	M	mAMES	6.89
Student	GM06	M	mAMEP	30.89
Student	GM06	M	mAMP	3.21
Student	GM06	M	mPTd	11.84
Instructor	GM06	M	mAMES	4.38
Instructor	GM06	M	mAMEP	28.05
Instructor	GM06	M	mAMP	1.39
Instructor	GM06	M	mPTd	9.09
Student	GM15	F	mAMES	2.08
Student	GM15	F	mAMEP	8.64
Student	GM15	F	mAMP	0.79
Student	GM15	F	mPTd	4.23
Instructor	GM15	F	mAMES	1.80
Instructor	GM15	F	mAMEP	8.13
Instructor	GM15	F	mAMP	0.81
Instructor	GM15	F	mPTd	2.56

**Table 5:** Mean muscle volumes (mm^3^) and coefficient of variation produced by investigators of varying expertise via the repeated surface mapping and frustrum modeling of four jaw muscles across two *A. pulchellus* specimens. (A) Male specimen (MUVC GM06). (B) Female specimen (MUVC GM15).

	A	mAMES	mAMEP	mAMP	mPTd	B	mAMES	mAMEP	mAMP	mPTd
Mean		5.63	29.47	2.30	10.46		1.94	8.39	0.80	3.40
Standard deviation		1.77	2.01	1.29	1.94		0.20	0.36	0.01	1.18
Coef. of variation		31.5	6.8	56.0	18.6		10.2	4.3	1.8	34.8

**Fig. 6:**
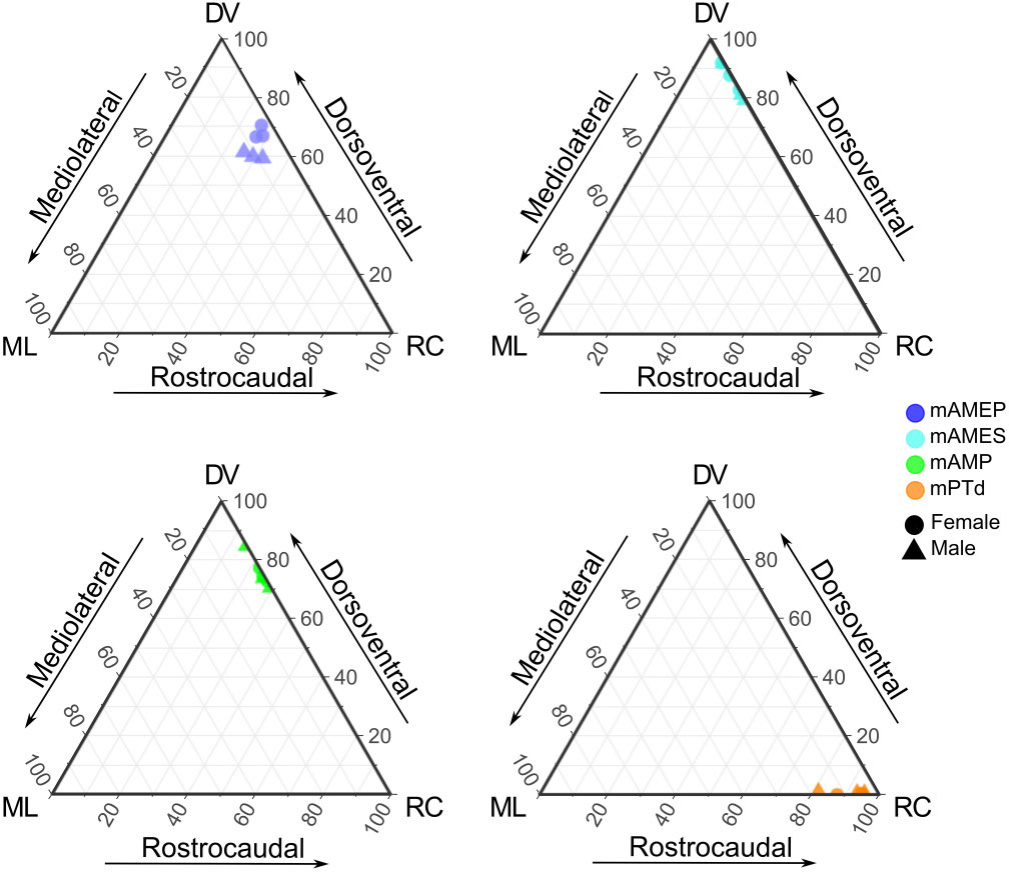
3D vectors of *Anolis pulchellus* jaw muscles projected into ternary space. Point alignment illustrates muscle orientation along different axes where DV = dorsoventral, RC = rostrocaudal, and ML = mediolateral. The surface areas and centroids of interest from each skull (MUVC GM06, GM09, PN14, GY09, GM10, GM15) were determined by individual investigators (see Table 2, Fig. 4). There are clear differences in muscle fiber orientation across the sexes for mAMEP only.

**Fig. 7:**
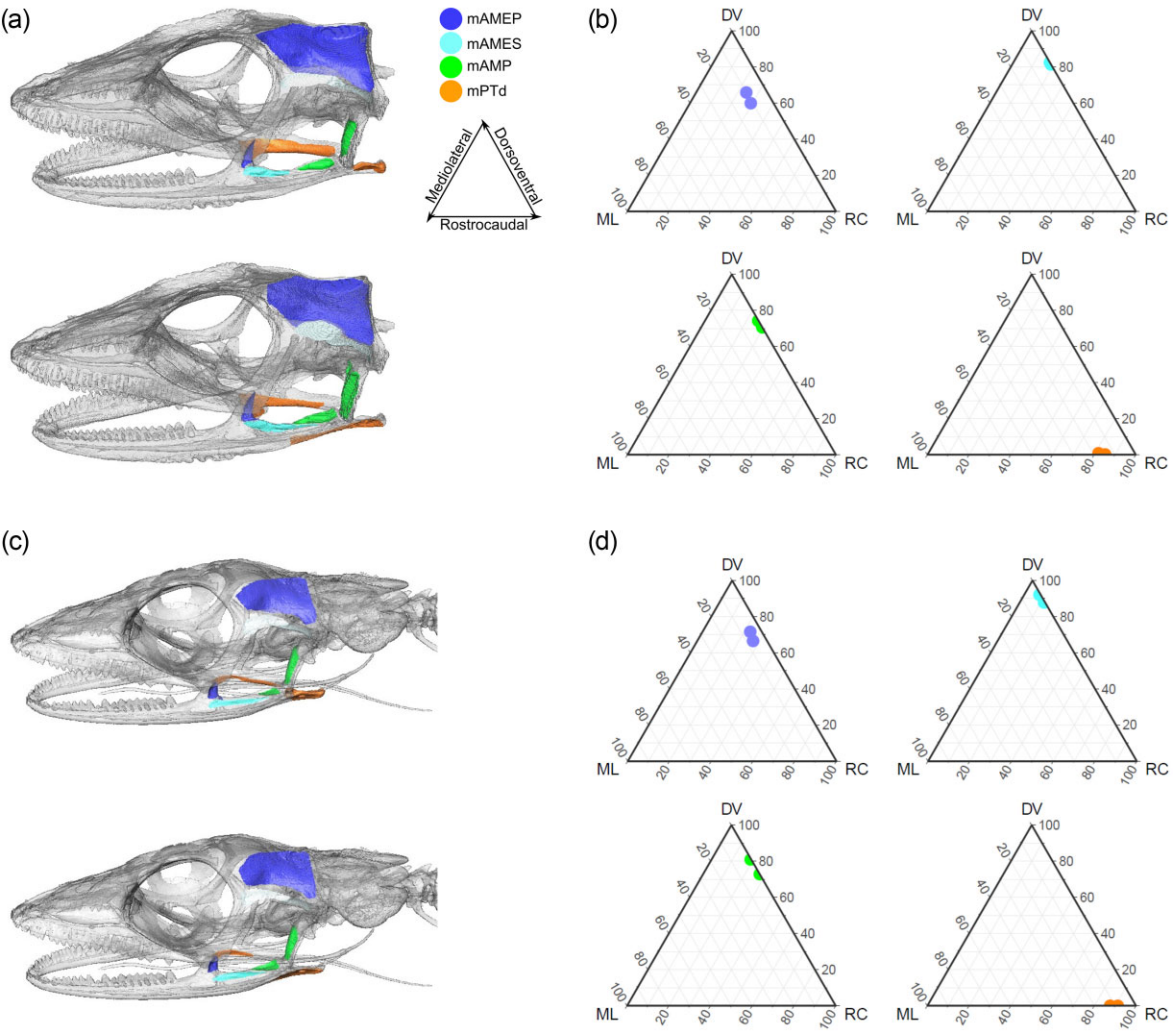
Repeat reconstructions of *Anolis pulchellus* jaw muscle attachment surface maps produced by two different pairs investigators. (a) Mapped muscle attachments for specimen MUVC GM06, male, produced by a single student and instructor pair. (b) 3D vectors of specimen MUVC GM06 jaw muscles projected into ternary space. (c) Mapped muscle attachments for specimen MUVC GM15, female, produced by a single student and instructor pair. (d) 3D vectors of specimen MUVC GM15 jaw muscles projected into ternary space. Point alignment illustrates muscle orientation along different axes. There is less variation between the mapped muscle attachments for each pair of skulls than within each individual skull.

When projecting each muscle into ternary space, there were no clear differences in muscle orientation between males and females, with the exception of mAMEP (Fig. 6). Despite this distinction, the fibers of mAMEP were predominately oriented along the dorsoventral axis, and to a lesser degree along the rostrocaudal axis, in both males and females. mAMES and mAMP had even stronger dorsoventral alignments, with mPTd being oriented almost entirely along the rostrocaudal axis, all regardless of sex (Fig. 6). These patterns were also observed in the replicate maps produced by the student-instructor pairs (Fig. 7).

#### Landmark analysis

**Fig. 8:**
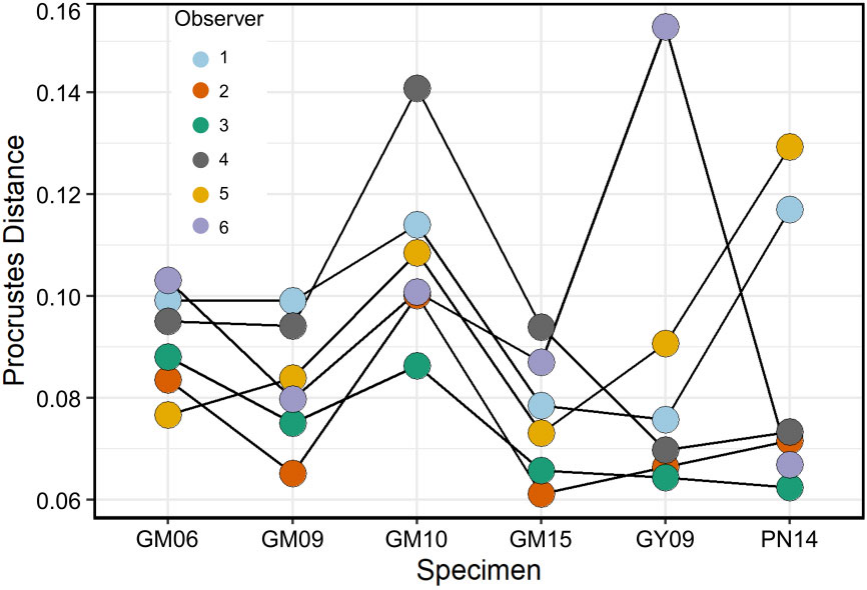
Procrustes distance for each specimen of *Anolis pulchellus* from the overall shape. Colors represent the observer who placed landmark points. There is considerable overlap between observers in the overall Procrustes distances for each specimen, indicating that observers were consistent in overall landmark placement, particularly in specimens MUVC GM06, GM09, and GM15.

The investigators (five students plus instructor FR McGechie) were consistent in their overall landmark placement, with considerable overlap between the Procrustes distances for each specimen (Fig. 8). The greatest amounts of variation occurred on specimens PN14 and GY09 and are likely due to student error in landmark placement, specifically, having placed landmarks that slightly clipped through portions of the skull, making the landmarks at slightly different depths on the bony surface.

The mean ICC across all specimens and observers was 0.862, indicating high reliability between trials. However, there is a range in ICC estimates between landmark parings. The lowest ICCs were from interlandmark distances involving the right jaw joint, the right palatobasal joint, the preotic pendant, and the left parietal which ranged from 0.136 to 0.391. The highest reliability was for landmarks involving the left postorbital and the left otic joint (0.999).

**Fig. 9:**
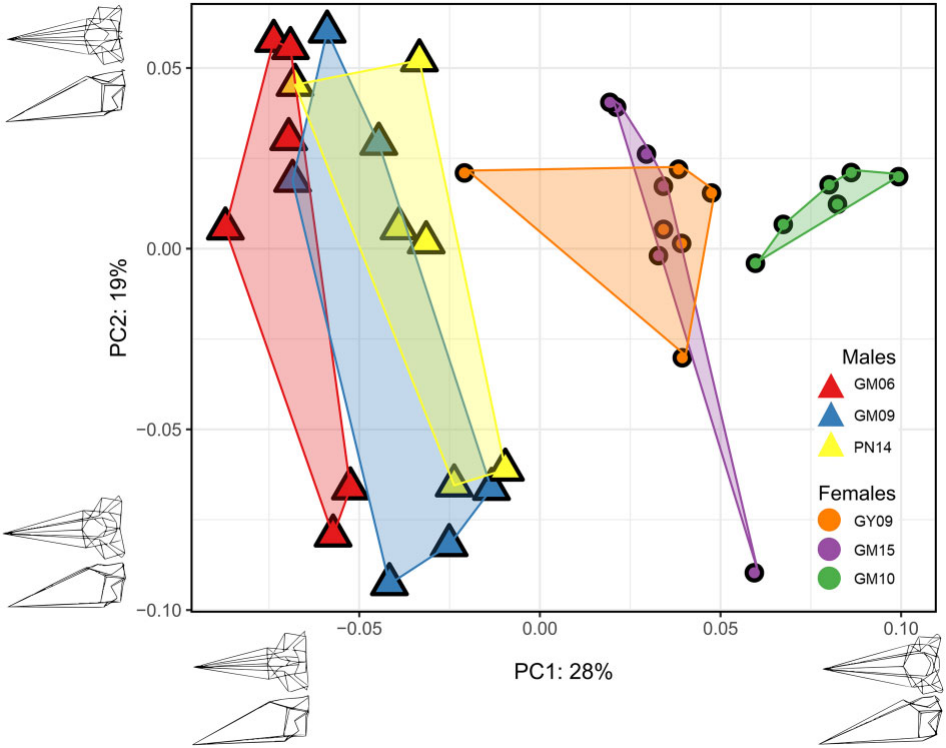
Results of principal components analysis on Procrustes coordinates of the overall cranial shape of the *Anolis pulchellus* specimens. PC1 (28%) separates the male (MUVC GM06, GM09, PN14) from female specimens (MUVC GY09, GM10, GM15) and is significantly correlated with logged centroid size (p < 0.05, r = 0.79). PC2 (19%) appears to be related to observers given the spread of replicates on the y-axis, except for in MUVC GY09 and MUVC GM10. The variation of the sample appears to be driven by points related to the parietal crest. For the most part, each specimen occupies its own distinct morphospace with little overlap, with the exception of the males (MUVC GM06, GM09, PN14), which do have more prominent parietal crests.

Principal component analysis on Procrustes coordinates of the overall cranial shape of the *A. pulchellus* specimens found that PC1 accounts for 28% of the variation observed and separates the specimens based on sex (Fig. 9). This separation is significantly correlated with logged centroid size (*P* < 0.05, r = 0.79). The lack of overlap observed between males and females is likely attributed to males having a more prominent parietal crest. PC2 accounts for 19% of the variation and separates the specimens according to the landmark placement of each investigator. In general, each specimen occupies its own distinct cluster in morphospace which is driven by points associated with the parietal crest. The landmark for Mid-Parietal Crest 1 accounted for much of the variation observed. Given that Mid-Parietal Crest 1 is a midpoint between two adjacent landmarks and not a distinct anatomical structure, it is subject to increased interpretation. Defining this region as a sliding curve semilandmark (Pelletier et al. 2022) may have helped to decrease some of the variation observed within the sexes. This example highlights the importance and potential impact of landmark choice on higher-order geometric morphometrics.

### Anolis sagrei

#### Muscle volume segmentation

**Fig. 10:**
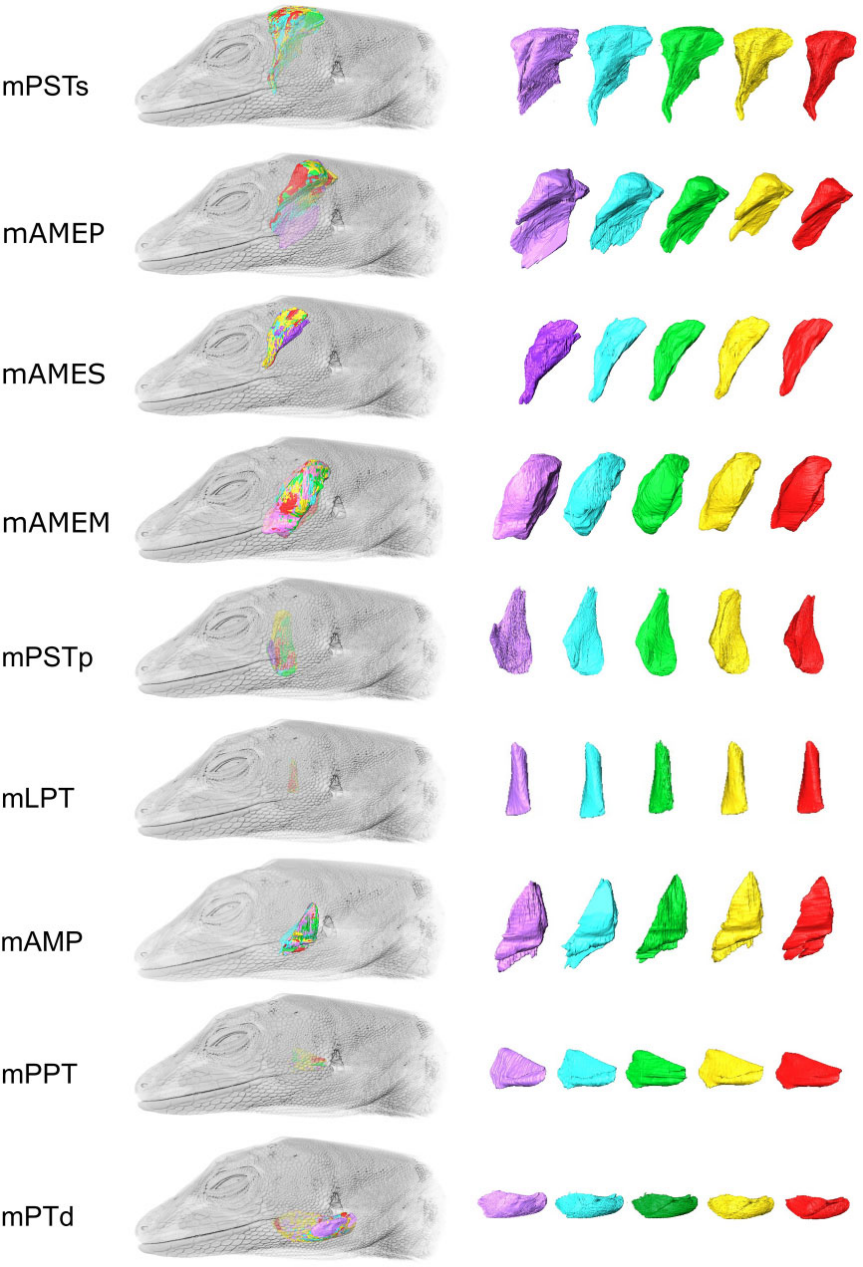
Variations in the interpretation of *Anolis sagrei* (MUVC LI100) jaw muscles. 3D models of muscle volumes produced by different investigators are superimposed over the same volume rendering of *A. sagrei*. Illustration of interobserver variation in the segmented volumes of each muscle is shown. Each color corresponds to a single investigator (see Table 6).

**Fig. 11:**
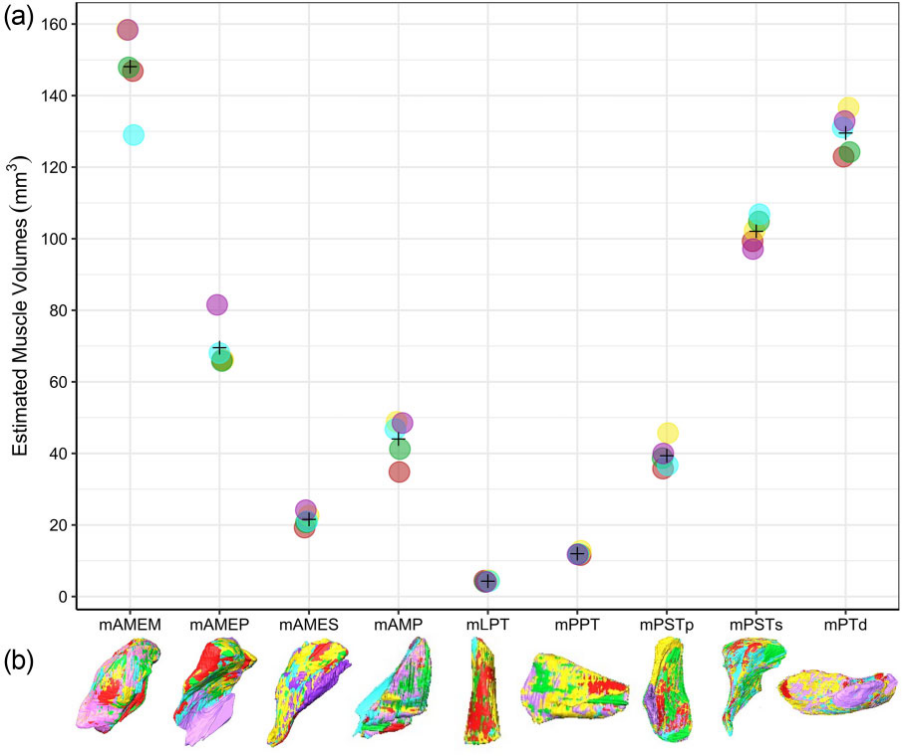
Interobserver variation in 3D segmented volumes of the same *Anolis sagrei* (MUVC LI100) jaw muscles. (a) Crosshatches illustrate mean estimated muscle volumes across all five investigators. (b) Overlaying 3D volumes of each investigator's segmented muscles. Colors correspond to the same-colored investigator as in Figure 10 and Table 6.

The segmented jaw muscle volumes produced by the different student investigators from the same specimen of *A. sagrei* (MUVC LI100) appear similar in general shape (Fig. 10) as well as in volume (Fig. 11, Table 6). The investigators had similar interpretations of the muscle bellies, with the bulk of the variation occurring along the outer boundaries of each muscle. This issue is particularly evident for mAMEM and mAMEP, which exhibited the greatest variation in segmentation. These muscles are poorly constrained by surrounding bony structures and have more diffuse fascial boundaries between neighboring muscles and were therefore subjected to more interpretation during segmentation. mAMEM has other muscles surrounding it with poor fascial separation whereas the rostral surface of mAMEP has poor fascial separation from mAMEM among other soft tissues. Other muscle boundaries were more straightforward resulting in less difference in interpretation among investigators, with mLPT and mPPT being the most consistent (Fig. 11) as they are clearly defined by fascia and surrounding nonmuscular tissues. Nonetheless, coefficient of variation in segmented muscle volumes were small compared to *A. pulchellus*, ranging from 4.0% to 13.6%. Muscle volumes were highly repeatable among observers with an ICC of 0.99, suggesting much greater variation among muscles than between observers.

**Table 6:** Mean muscle volume estimates and coefficient of variation produced via the repeated segmentation of a single *A.sagrei* specimen by five different investigators. Investigator colors correspond to those in Figs. 10 and 11.

			Muscles volumes (mm^3^)								
Investigator	Taxon	Specimen	mAMES	mAMEM	mAMEP	mAMP	mPSTs	mPSTp	mPT	mLPT	mPPT
	*Anolis sagrei*	MUVC LI100	24.13	158.06	81.52	48.50	97.11	39.97	132.88	4.10	11.77
	*Anolis sagrei*	MUVC LI100	21.04	128.99	68.04	46.78	106.85	36.70	131.06	4.30	11.86
	*Anolis sagrei*	MUVC LI100	20.73	147.89	65.82	41.20	104.83	38.68	124.22	4.09	11.84
	*Anolis sagrei*	MUVC LI100	22.61	158.37	66.14	48.83	102.29	45.68	136.61	4.53	12.79
	*Anolis sagrei*	MUVC LI100	19.26	146.85	66.17	34.80	99.29	35.75	122.91	4.44	11.58
		Mean	21.55	148.03	69.54	44.02	102.07	39.35	129.54	4.29	11.97
		Standard deviation	1.87	12.02	6.67	5.99	3.96	3.90	5.83	0.20	0.47
		Coef. of variation	8.7	8.1	9.7	13.6	3.9	9.9	4.5	4.7	4.0

#### Muscle mapping

**Fig. 12:**
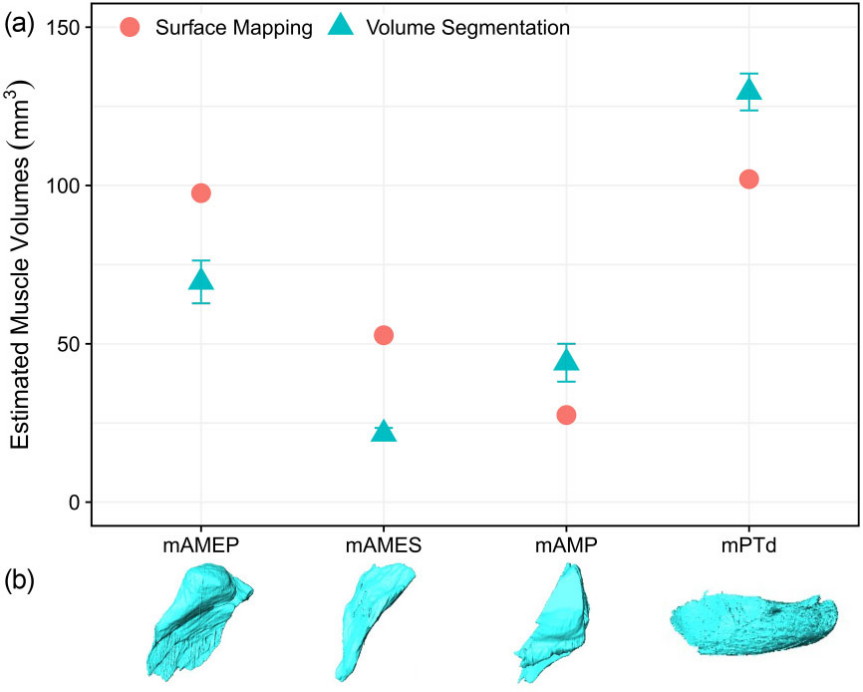
Comparison of methods used to estimate volumes of *Anolis sagrei* (MUVC LI100) jaw muscles. (a) Triangles represent an average of five trials (see Table 6) of segmented DiceCT data with standard deviation. Circles represent a single trial based on surface attachment muscle mapping and frustrum modeling (see Table 7). Coefficient of variation between estimates: mAMEP (17.5), mAMES (48.0), mAMP (20.9), and mPTd (9.9). (b) Illustration of each muscle's general shape when in left lateral view produced using 3D volume segmentation.

The jaw muscle volumes estimated for *A. sagrei* (MUVC LI100) using mapped surface attachments and frustrum modeling showed some variation from the volumes estimated using volume segmentation (Fig. 12, Table 7). The differences in muscle volume estimates are likely attributed to unclear margins of bony muscle attachments, particularly in the case of mAMES and its insertion and origin on the surangular and squamosal, respectively, which lack clear osteological correlates of muscle attachment (Holliday 2009). Our interpretation of these muscles included bellies in the segmentation that were not well represented by their bony attachments. Despite this, the difference between the two methods is small with a coefficient of variation between estimates of 48.0% for mAMES, 20.9% for mAMP, 17.5% for mAMEP, and 9.9% for mPTd.

**Table 7:** Estimated muscle volumes produced via the surface mapping and frustrum modeling of four jaw muscles of *A. sagrei* (MUVC LI100) and the coefficient of variation between muscle volumes produced via volume segmentation of the same *A. sagrei* specimen.

Taxon	Muscle	Volume (mm^3^)	Coef. of variation
*Anolis sagrei*	mAMEP	97.6	17.5
*Anolis sagrei*	mAMES	52.7	48.0
*Anolis sagrei*	mAMP	27.5	20.9
*Anolis sagrei*	mPTd	102	9.9

#### Muscle fascicle architecture

**Fig. 13:**
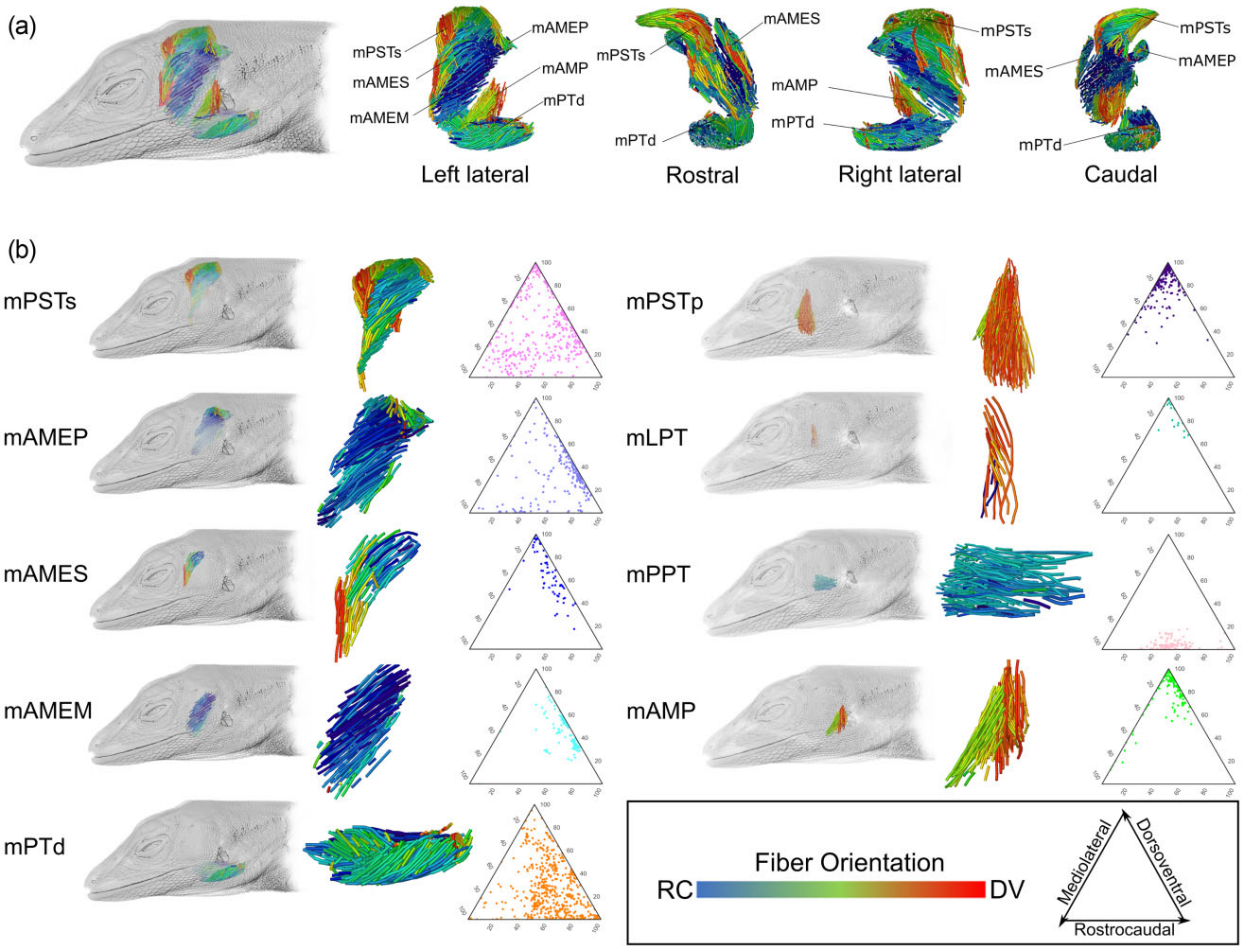
3D fascicle architecture of *Anolis sagrei* (MUVC LI100) jaw muscles. (a) Combined jaw musculature of *A. sagrei*. Fascicle orientation along the dorsoventral (DV) and rostrocaudal (RC) axes are represented by color, as shown. (b) 3D fascicle models of isolated jaw muscles shown independently as well as superimposed over a volume rendering of *A. sagrei* in left lateral view. Each model is accompanied by a ternary plot comprised of their fascicle vectors.

The internal architecture of *A. sagrei* (MUVC LI100) jaw muscles produced using Avizo Xfiber illustrate clear patterns in fascicle length and orientation that approach the visible architecture from the DiceCT data (Fig. 13). In addition, patterns of muscle pennation and fiber orientation in the autosegmented 3D fascicle models are easily observed when plotted in ternary space (Fig. 13b). Muscles with largely parallel fascicles had a fiber orientation clustered along a single axis in ternary space, such as mAMEM, mLPT, and mPPT. Pennate muscles, muscles with non-parallel fiber orientations, demonstrated clusters of fibers in multiple orientations along the different axes, such as mPSTs, mPTd, and mAMEP. Due to their comparative complexity, pennate muscles were susceptible to errors during the automation process resulting in ternary plots containing more noise artifacts (Fig. 13b).

## Discussion

The current study presents advances in the digital imaging and 3D modeling of *Anolis* heads with a focus on the jaw musculature and neighboring structures of two ecomorphs, *A. pulchellus* and *A. sagrei*. Anoles are model organisms for studies of adaptive evolutionary processes due to their abundance of morphological, behavioral, and ecological diversity (Losos 2009; Legreneur et al. 2012).

### Anolis pulchellus

Our analysis of the cranial structures of *A. pulchellus* found evidence for strong sexual dimorphism with regard to skull size, which is directly supported by the literature (Losos 2009; Sanger et al. 2013). Throughout the clade, males exhibit elongated snouts compared to females and this differentiation typically solidifies early in development (Sanger et al. 2013). This change in male cranial size as opposed to shape is thought to be associated with the need for elevated bite forces during intrasexual competition where males will bite one another in territorial fights (Calsbeek and Marnocha 2006).

Our estimates of muscle volumes for *A. pulchellus* show males averaging substantially larger muscles, in particular the temporal muscle mAMEP, than females. This increase in muscle is likely to result in the production of comparatively greater bite forces which are associated with male-male combat not just in anoles, but in species across the Iguanian clade (Husak et al. 2006; Lappin et al. 2006; Jones et al. 2020). Larger muscle volumes can require larger bony surface areas for attachment, which is also illustrated in our findings as male *A. pulchellus* specimens also have larger parietal crests compared to their female counterparts. Taken together, our findings support the notion that male intraspecific competition may be a driving factor of musculoskeletal dimorphism in the heads of anoles. In addition, the greatest amounts of variation measured in our sample of *A. pulchellus* resulted from differences in complex, sexually dimorphic skull traits and landmark selection rather than interobserver error. This is distinct from what was found in Shearer et al. (2017), in which the most prominent sources of variation in metric data were found among observers (i.e., interobserver error was the highest source of combined error). Further, Shearer et al. (2017) suggest that training observers in the placement of landmarks on the relevant morphology may assist in reducing interobserver error. All observers were trained in the placement of landmarks and the morphology of the *Anolis* skull as a result of the PTH_AS 8285 (*3D Imaging and Computational Biology for Research Applications*) course, which could have alleviated the potential for higher interobserver error. The similar training among investigators lends credibility to the accuracy of our 3D analyses as well as reinforcement of the notion put forth by Shearer et al. (2017) that training, discussion, or even an atlas of landmarks could help reduce interobserver error in studies with multiple observers or combined datasets.

### Anolis sagrei

The current study demonstrates the ease, reliability, and accuracy with which *A. sagrei* jaw architecture can be segmented and visualized using high-quality digital contrast imaging. As with dissection-based observations of anatomy, interpretation cross-sectional DiceCT data require a familiarity with the morphological structures being observed and their relation to the surrounding hard and soft tissues. Even for experienced anatomists, it can be difficult to untangle such structural relationships in 3D, making the misidentification of whole or partial structures possible. Here in the case of jaw muscles within the adductor chamber of *A. sagrei*, we expected muscles lacking clear fascial planes and/or physical separation of bony muscle attachments to exhibit the greatest amounts of interobserver variation in their interpretation. While simplified, the 3D models produced by our investigators largely conformed to the known condition of anole jaw muscles indicated in the literature (Daza et al. 2011; Wittorski et al. 2016), and the measurement of error between investigators with regard to individual muscle volume estimates is minimal.

The jaw muscle volumes reported here for *A. sagrei* are expected to be minimally smaller than a true volume of fresh *Anolis* jaw muscle tissue save expect ∼5% shrinkage from fixation and preservation. Iodine and fixation solutions are known to cause shrinkage of tissues (Early et al. 2020); however, the tissue shrinkage reported in previous studies of DiceCT-based shrinkage artifacts used excised samples of skeletal muscle removed from its bony attachments (Vickerton et al. 2013; Green et al. 2019). Thus, it is not surprising that these previous studies found substantial differences in tissue volumes as the muscle fibers contracted in an isolated, untethered environment. Other studies have reported shrinkage artifacts in neural tissues, isolated smooth muscle, or glandular organs (Hedrick et al. 2018; Gignac and Kley 2018; Dawood et al. 2021) all of which behave differently from skeletal muscles with their attachments in place.

Avizo Xfiber's interpretation of muscle fiber architecture captures structural complexity with 3D analysis. This automated method offers significant benefit over the hand segmentation of muscle fibers, particularly in its time savings as well as the fact that it captures all or most fascicles, rather than just a few individual fascicles. The time investment required to train investigators in the hand segmentation of muscle fiber bundles would be costly and potentially just as error-prone as investigators in our study and other authors have found it easy to slip from one fascicle to a neighbor while attempting to track them through planes of the data. As automated fascicle tracking technology continues to develop (e.g., Sullivan et al. 2019; Dickinson et al. 2019; Katzke et al. 2022, Arbour 2023), one can expect improvements in automation and data visualization that will result in better representations of the anatomy. In the interim, comparisons of our 3D reconstructed data to data collected through classical means (i.e., dissection and histology) are necessary for more in-depth analyses of muscular form and function in anoles and other animals.

## Conclusion

Throughout this study we demonstrated the accessibility of emerging 3D modeling and computational approaches while identifying sources of potential error in their analytical prowess. Further development and implementation of these methods holds the potential for morphologists to collect and share accurate, digital, 3D anatomical data in a manner previously unprecedented. Our contributions to the visualization of anole musculoskeletal anatomy hold the potential to lend to future studies of cranial performance, feeding behavior, and evolution across the clade.

## Data Availability

Data underlying this article are hosted on MUVC or Hollidaylab spaces on Morphosource and Sketchfab.
